# Mathematical modeling and optimizing the *in vitro* shoot proliferation of wallflower using multilayer perceptron non-dominated sorting genetic algorithm-II (MLP-NSGAII)

**DOI:** 10.1371/journal.pone.0273009

**Published:** 2022-09-09

**Authors:** Fazilat Fakhrzad, Abolfazl Jowkar, Javad Hosseinzadeh

**Affiliations:** 1 Department of Horticultural Science, College of Agriculture, Shiraz University, Shiraz, Iran; 2 Department of Electrical Engineering, Amirkabir University of Technology, Tehran, Iran; National University of Kaohsiung, TAIWAN

## Abstract

Novel computational methods such as artificial neural networks (ANNs) can facilitate modeling and predicting results of tissue culture experiments and thereby decrease the number of experimental treatments and combinations. The objective of the current study is modeling and predicting *in vitro* shoot proliferation of *Erysimum cheiri* (L.) Crantz, which is an important bedding flower and medicinal plant. Its micropropagation has not been investigated before and as a case study multilayer perceptron- non-dominated sorting genetic algorithm-II (MLP-NSGAII) can be applied. MLP was used for modeling three outputs including shoots number (SN), shoots length (SL), and callus weight (CW) based on four variables including 6-benzylaminopurine (BAP), kinetin (Kin), 1-naphthalene acetic acid (NAA) and gibberellic acid (GA_3_). The R^2^ correlation values of 0.84, 0.99 and 0.93 between experimental and predicted data were obtained for SN, SL, and CW, respectively. These results proved the high accuracy of MLP model. Afterwards the model connected to Non-dominated Sorting Genetic Algorithm-II (NSGA-II) was used to optimize input variables for obtaining the best predicted outputs. The results of sensitivity analysis indicated that SN and CW were more sensitive to BA, followed by Kin, NAA and GA. For SL, more sensitivity was obtained for GA_3_ than NAA. The validation experiment indicated that the difference between the validation data and MLP-NSGAII predicted data were negligible. Generally, MLP-NSGAII can be considered as a powerful method for modeling and optimizing in vitro studies.

## Introduction

*Erysimum cheiri* (L.) Crantz, commonly named wallflower, is a biennial or perennial ornamental plant which belongs to the Brassicaceae family. Wallflowers are grown all over the world in a variety of colors as an important bedding and garden flower [1, 2]. This species is widely used as a popular landscape plant, flowering pot plant, and also as a rock garden flower [2]. A number of cardiotonic glocosides have been detected from various organs of *E*. *cheiri*. It has also been extensively used as cardioactive, antifissure and anti-inflammation, emmenagogue, fertilizer and anti-tumor in the traditional medicine [3]. Due to the increasing demands of the market for the valuable ornamental plant, conventional propagation and breeding approaches are no longer sufficient and it is necessary to establish high quality biotechnological methods. Plant tissue culture is an important agricultural biotechnology technique that provides the production of crops with uniform characteristics in a short time and cost-effective systems under aseptic conditions [4]. There is no report for micropropagation of *Erysimum cheiri* so a robust and efficient protocol has yet to be fully developed. Therefore, the use of breeding methods and biotechnological techniques in this plant is encountered to some limitations. Several intrinsic factors (e.g., genotype, organ type, and explant developmental age) and also external parameters (e.g., vitamins, plant growth regulators (PGRs), carbohydrate source, temperature, and light) delimit in vitro shoot growth and development [5].

PGRs play a vital role for *in vitro* organogenesis such as shoot proliferation [6, 7], shoots organogenesis [8], somatic embryogenesis [9], and callus induction [8, 10]. Therefore, finding the optimized amount of media compositions for achieving ideal results is a versatile challenge for *de novo* plant micropropagation [4, 11]. Since the traditional analytical methods such as linear regression are not suitable for non-linear biosystem [7, 12], artificial neural networks (ANN) as the non-linear modeling techniques have become a reliable method to predict and optimize the correlations between the input and output of a biological process [12, 13]. ANNs include of numerous highly interconnected processing neurons that work in parallel to find a solution for a specific problem [14]. ANNs are learned by examples. The examples should be intently chosen otherwise the model might be working inaccurately [14, 15]. ANNs are able to recognize the relationship between output and input variables and identify the inherent knowledge existent in the datasets without previous physical considerations. Hence, ANNs are considered as a “black box” [16, 17]. Multilayer Perceptron (MLP), is one of the common artificial neural networks (ANNs) applied for modeling and predicting *in vitro* culture processes [4]. MLP is inspired by the neural structure of the human brain and consists of an input layer, one or more hidden layers, and an output layer [14]. Like the human neural network, ANNs contain nodes, each of which receives a number of input variables and produce a single target variable that is a relatively simple function of the input variables [18]. The connections are based on weights given by values that were defined in the training process so that the output values will be as similar as possible to the values that were obtained from the training model. Network fitting is conducted by means of the back-propagation algorithm, which estimates the weights through the connections that are performed in the opposite direction of the subsequent layer [14, 16]. Recently, a number of reports have been published about the use of artificial intelligence models in plant tissue culture procedures [4, 12, 16, 18–23].

Evolutionary optimization algorithms are considered the powerful mathematic methods for solving complex, multidimensional problems such as designating optimal factors for micropropagation with high speed and accuracy [4]. There are different types of evolutionary optimization algorithms and genetic algorithm (GA) has been applied to the vast majority of plant tissue culture optimization studies relating to shoot proliferation [17], secondary metabolite production [23], and somatic embryogenesis [21]. GA is an optimization algorithms based on the principles of genetic variation and natural selection. It evolves finding the best solution for a specific problem [16]. Plant tissue culture is a multi-objective system may not be optimized using GA as a single-objective function, therefore multi-objective algorithms are necessary for the optimization of outputs [19]. Classical optimization methods, including multi-criterion decision making methods, have established a model for converting multi- objective optimization to a single-objective optimization issue through emphasizing one particular Pareto-optimal solution at a time. In this method, multiple runes are required to obtain different possible solutions [20]. One of the first evolutionary multi-objective optimization algorithms, which is useful for finding the solution domain in order to detect Pareto-optimal solutions within a multi-objective scheme is known as the Non-dominated Sorting Genetic Algorithm-II (NSGA-II) [20]. A few reports used ANN-NSGA-II for predicting and optimizing plant sterilization [20], shoot proliferation [19] and somatic embryogenesis [21] of *Chrysanthemum × grandiflorum*. Hesami et al. (2019) [20] used MLP- NSGAII to achieve the highest efficiency and optimum concentrations of disinfectants as well as immersion times to minimize *in vitro* contamination frequency (CF) and maximize the explant viability (EV) of *Chrysanthemum*. The R^2^ (over 94%) indicated that MLP-NSGAII was a powerful model for optimizing and forecasting *in vitro* sterilization of *chrysanthemum*. They also suggested that MLP-NSGAII can be employed as a precise method for different areas of *in vitro* culture. They also applied the ANFIS linked to NSGAII to optimize the appropriate hormonal combinations (2,4-D and BAP), carbohydrate (sucrose, fructose, and glucose) and light quality and further maximize the embryogenesis frequency (EF) and number of somatic embryogenesis (NSE) in *chrysanthemum*. They reported a high efficiency and accuracy of ANFIS- NSGAII on the modeling of the somatic embryogenesis (R^2^> 0.92) [21]. In another study [19], RBF-NSGAII was used to model and predict the optimal levels of BAP, IBA, PG and sucrose on shoot proliferation parameters in order to maximize the shoot number and shoot length and concurrently minimize the callus weight of *chrysanthemum*. High R^2^ (> 0.76) between observed and predicted values indicated that RBF-NSGAII can be considered as an efficient computational strategy for modeling and optimizing *in vitro* organogenesis [19].

In this study, we tried to propose a model for shoot proliferation by using non-linear MLP- NSGAII modeling and optimization procedure. In this way, making a strong link between the MLP model and NSGAII was our first priority in order to find the highest efficiency and the optimum concentrations of PGRs for significant *in vitro* shoot proliferation. Generally, the objective of this study is to model and optimize the appropriate plant growth regulators’ compositions for maximum shoot proliferation of *E*. *cheiri*.

## Materials and methods

### Plant materials

Nodal segments (1.5 cm) were harvested from the tetraploid wallflower plants kept in the greenhouse. After washing with tap water for 30 minutes, the explants were placed in a solution of detergent and water (1:1) and washed. Subsequent disinfection steps were performed under a laminar airflow chamber by soaking the explants in 70% ethanol for 30 seconds followed by 3% sodium hypochlorite solution for 7 minutes. The explants were washed three times with sterile distilled water and then put in MS medium [24] containing 6% agar and 3% sucrose. The pH was adjusted to 5.8 using 1 N HCl or 1 N NaOH before autoclaving at 121°C. The cultured flasks were exposed to 16/8 h (light/dark) photoperiod for 4 weeks with a light intensity of 80 umol.m^-2^.s^-1^ and a temperature of 24 ± 2°C.

### Experimental design

This experiment was set up in a completely randomized design (CRD) with the factorial arrangement and four replicates, each containing four explants. The explants were inoculated in the proliferation medium including different concentrations of 6-benzyladenine (BA) (0, 0.5, 1, 2 mg.l^-1^), kinetin (Kin) (0, 0.5, 1, 2 mg.l^-1^), naphthaleneacetic acid (NAA) (0, 0.1 mg.l^-1^) and gibberellic acid (GA_3_) (0, 0.2 mg.l^-1^). The shoots number (SN), shoots length (SL), and callus weight (CW) were determined after 8 weeks of culture.

### Multilayer perceptron (MLP) model

**Fig 1:**
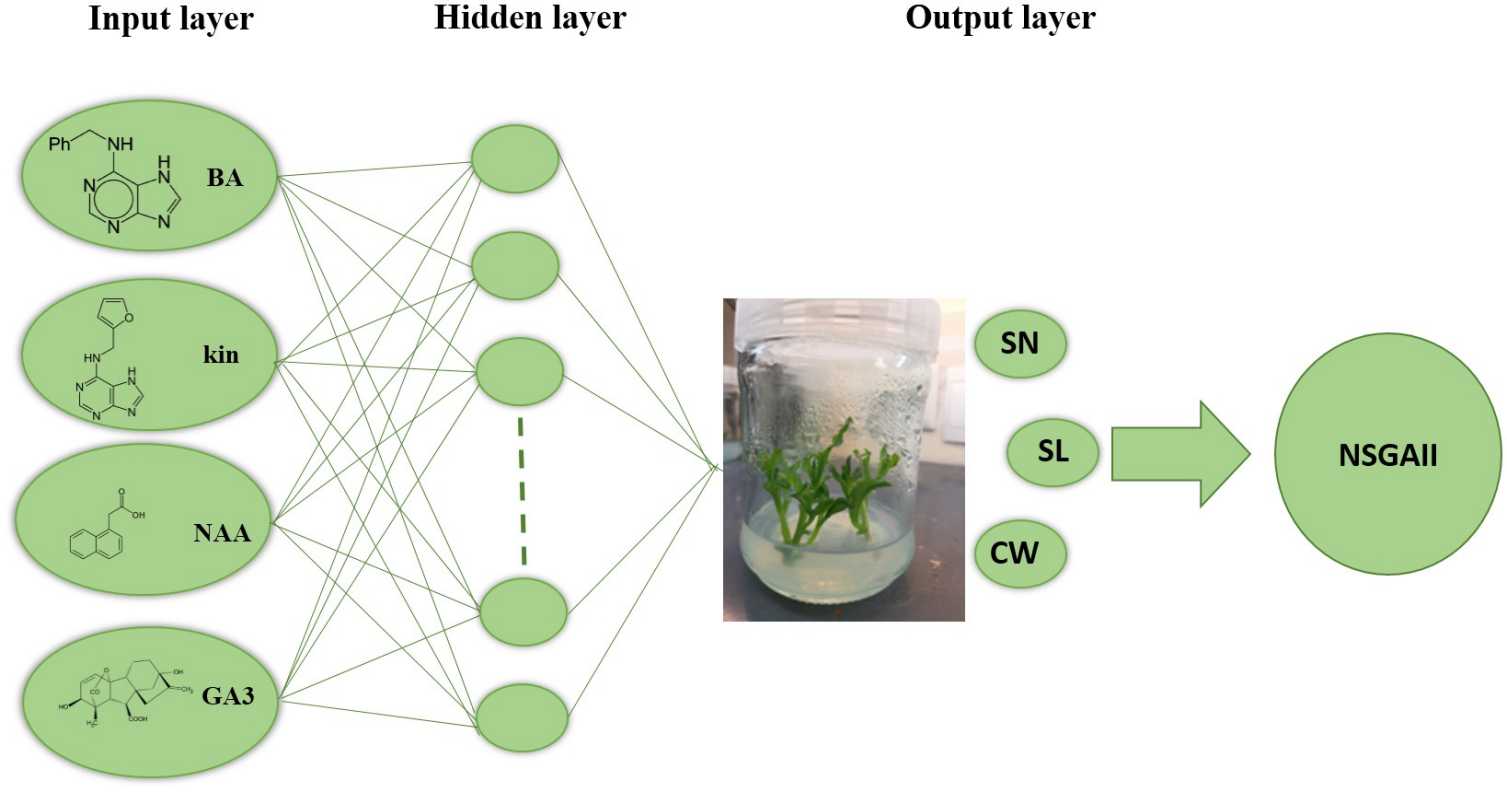
The schematic diagram of the proposed MLP method.

In order to construct the MLP model, four types of PGRs were used as inputs and SN, SL, and CW were considered as outputs for the modeling of *in vitro* proliferation (Fig 1). MLP was applied for obtaining the maximum rate of SN and SL as well as the minimum rate of CW. Prior to modeling, the data were randomly divided into 80% training and 20% testing sets. The datasets of input and output were normalized between -1 and 1 by mapminmax transformation. To detect outliers, principal component analysis (PCA) was used; however, no outlier was identified. This model provides inputs and outputs to the network by a supervised training procedure, while the training process continues until the following function would be minimized:

$$E=\frac{1}{K}\sum_{k=1}^{k}{\left(y_{k}-{\hat{y}}_{k}\right)}^{2}$$

Where *K is* the number of data, *yk* is the *kth* observation output, and ${\hat{y}}_{k}$ is the *kth* predicted output. In a three-layer MLP with *m* neurons in the hidden layer and *n* input variables, $\hat{y}$ is calculated as:

$$\hat{y}=f\left[\sum_{j=1}^{m}w_{j}.g\left(\sum_{i=1}^{n}w_{ji}x_{i}+w_{j0}\right)+w_{0}\right]$$

where *wj*: weight that connects the *jth* neuron of hidden layer and neuron of output layer, *wji*: the weight connecting the *ith* input variable and *jth* neuron of hidden layer, *xi*: the *ith* input variable, *wj*0: bias of the *jth* neuron of hidden layer, *w*0: bias related to the output neuron, *g*: the transfer functions for hidden layer, and *f*: transfer functions for the output layer.

In this study, three-layer perceptrons (feed forward back-propagation network) was applied with hyperbolic tangent sigmoid (tansig) and linear (purelin) for hidden and output layers transfer functions, respectively. A Bayesian Regulation was used for training of the network and determining the optimal weights and bias. Since the number of hidden units and the number of neurons in each node play an important role in the efficiency of MLP, they should be determined. There are some reports which show the optimal number of neurons in the hidden layer by means of some equations [4, 25], but they ultimately should be obtained by using trial and error. The large or low number of them results in under-fitting or over-fitting, respectively. In the current investigation, trial and error-based approach was used to detect the optimal neuron number in the hidden layer.

### Performance measures

Three MLP -model was trained for each of the three outputs including SN, SL, and CW. The best fitness for each model was determined based on the mean bias error (MBE), root mean square error (RMSE) and coefficient of determination (R^2^) as follows:

$$R^{2}=1-\frac{\sum_{i=1}^{n}{\left(y_{i}-\hat{y_{i}}\right)}^{2}}{\sum_{i=1}^{n}{\left(y_{i}-\overset{-}{y_{i}}\right)}^{2}}\;0\leq R^{2}\leq 1$$

$$RMSE=\sqrt{\left(\sum_{i=1}^{n}{\left({\hat{y_{i}}-y}_{i}\right)}^{2}\right)/n}\;0\leq RMSE\leq \infty$$

MBE=1n∑i=1nyi-y^i-1≤MBE≤+1

Where *n* is the number of data, *yi* is the value of observed datasets, and ${\hat{y}}_{i}$ is the value of predicted datasets and $\overset{-}{y_{i}}$ is the mean of observed values. RMSE and MBE values closer to 0, and R^2^ values closer to 1, show best performance of the constructed models [4, 12, 26]. The higher R^2^ and lower RMSE and MBE indicated better performance of the designed models [15].

### Optimization algorithm NSGA-II

**Fig 2:**
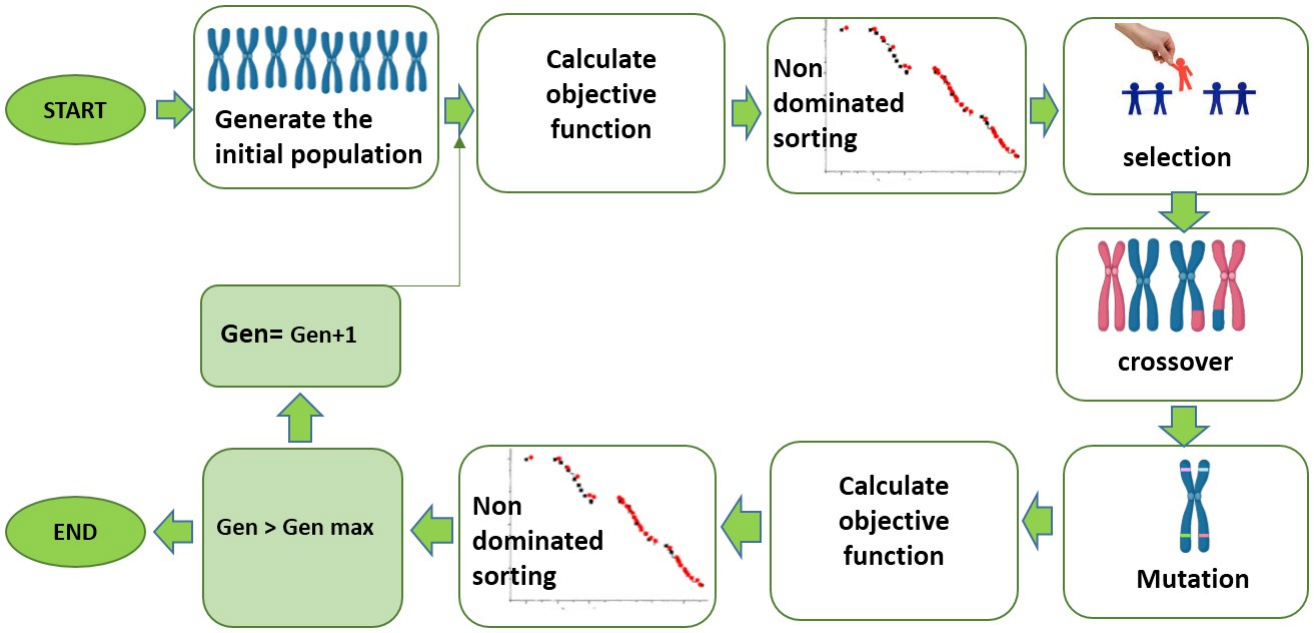
The schematic diagram of NSGAII optimization process.

The developed MLP model, as the fitness function, was subjected to additional practice using NSGA-II to determine the optimum amounts and combinations of input variables to achieve the best values of outputs (Fig 2). This algorithm first generates a number of random solutions and then the objective function is calculated for each solutions. The search for optimal solutions during NSGA-II implementation was limited to the lower and upper bounds of the input variables [20]. The binary tournament operator was used to select elite populations for crossover, based on two criteria: non-dominated sorting and crowding distance, which are two characteristics of a good pareto front. A mutation operator was applied to protect the algorithm from getting stuck in the local optimum. When the refining solutions are determined, the objective function values were recalculated and continued until one of the terminated criteria were attained [4]. In each generation, non-dominated solutions in objective space constitute a Pareto front; any point on this front can be an optimal solution of the problem [4].

We considered SN, SL and CW as three objective functions to recognize the optimum values of inputs based on the results of MLP Model. In this study, 50 initial population, 800 generation number, 0.8 crossover rate and 0.01 mutation rate were set. An ideal point on the pareto front is calculated so that while SN and SL were maximized and CW was minimized, the solution obtained from the following equation is minimized.

$$E=\sqrt{{\left(shn-n\right)}^{2}+{\left(SHL-l\right)}^{2}+{\left(CW-c\right)}^{2}}$$

Where *n* and *l* are the maximum SN, and SL respectively, and *c* is the minimum CW in the observed data. Objective function values were scaled between 0 and 1 before applying.

### Sensitivity analyses

Sensitivity analysis was applied on the obtained ANN model to determine the importance of input variables in the model. The sensitivity of SN, SL and CW against the applied PGRs) BA, Kin, NAA and GA_3_) is recognized by criteria including Variable Sensitivity Error (VSE): overall performance of the developed ANN-GA model if the certain independent variable is not available, and Variable Sensitivity Ratio (VSR) value: indicates the correlation between the VSE and the error of the ANN model when all variables are available. A more important variable indicates higher value of VSR. Input variables can be ranked based on their VSR value or importance [26]. MATLAB software (Matlab, 2010) was employed to write codes and run the model.

### Validation experiments

In order to evaluate the efficiency of the MLP-NSGAII, optimized hormonal combinations of shoot proliferation parameters (i.e., SN, SL, and CW) were tested experimentally.

## Results

In this study, the effects of four PGRs on shoot number (SN), shoot length (SL), and callus weight (CW) of *E*.*cheiri* were studied. The combination of two cytokinins BA and Kin showed that the concentrations of 2 mg.l^-1^ BA+ 2 mg.l^-1^ Kin and 2 mg.l^-1^ BA+ 1 mg.l^-1^ Kin produced the highest shoot length (3.90 cm and 3.86 cm, respectively), while the highest callus weight (0.25g) was formed in 2 mg.l^-1^ BA+ 2 mg.l^-1^ Kin. Also the combination of 1 mg.l^-1^ BA + 2 mg.l^-1^ Kin and 2 mg.l^-1^ BA + 1 mg.l^-1^ Kin produced more number of shoots (6.06 and 5.81, respectively). No SN, SL and CW were observed on MS medium without PGRs (control treatment). Applying 2 mg.l^-1^ BA + 0.1 mg.l^-1^ NAA in the absence of other hormones showed the highest shoot length (3.39 cm) and callus weight (0.08 g). The most frequent shoot number (4.44 and 4.75) was obtained in 2 mg.l^-1^ BA alone, or in combination with 0.1 mg.l^-1^ NAA. Also the interaction of BA as a cytokinin and GA_3_ was analyzed. The treatment 2 mg.l^-1^ BA alone and along with 0.2 mg.l^-1^ GA_3_ produced highest shoot number (4.44 and 4.63, respectively). The results of the combination of four used PGRs show that highest shoot number (6.5) and length (3.99 cm) were achieved in MS medium supplemented with 1 mg.l^-1^ BA, 2 mg.l^-1^ Kin, 0.1 mg.l^-1^ NAA and 0.2 mgl^-1^ GA_3_ (Table 1). The highest weight of callus (0.3 g) was observed in 2 mg.l^-1^ BA, 2 mgl^-1^ Kin, 0.1 mg.l^-1^ NAA and 0.2 mg.l^-1^ GA_3_ and minimum amount of callus (0 g) was gained in the control treatments (Table 1).

**Table 1:** Effects of BA, Kin, NAA, and GA_3_ on shoot number (SN), shoot length (SL), and callus weight (CW) of *E*. *cheiri*. Values in each column represent means ±SE.

BA	Kin	NAA	GA_3_	SN	SL(cm)	CW(g)
0	0	0	0	0.00 ± 0. 000	0.00 ± 0. 000	0.00 ± 0.000
0	0	0	0.2	0.00 ± 0. 000	0.00 ± 0. 000	0.00 ± 0. 000
0	0	0.1	0	0.00 ± 0. 000	0.00 ± 0. 000	0.00 ± 0. 000
0	0	0.1	0.2	0.38 ± 0.180	0.23 ± 0.104	0.00 ± 0. 000
0	0.5	0	0	1.37 ± 0.125	1.79 ± 0.006	0.00 ± 0. 000
0	0.5	0	0.2	2.38 ± 0.125	1.96 ± 0.004	0.00 ± 0. 000
0	0.5	0.1	0	2.25 ± 0.171	1.94 ± 0.003	0.00 ± 0. 000
0	0.5	0.1	0.2	2.50 ± 0.158	2.19 ± 0.006	0.00 ± 0. 000
0	1	0	0	2.69 ± 0.176	2.55 ± 0.007	0.00 ± 0.000
0	1	0	0.2	2.94 ± 0.193	2.76 ± 0.012	0.00 ± 0.000
0	1	0.1	0	3.00 ± 0.224	2.68 ± 0.007	0.00 ± 0.000
0	1	0.1	0.2	3.19 ± 0.164	2.89 ± 0.007	0.01 ± 0.003
0	2	0	0	4.38 ± 0.180	3.28 ± 0.004	0.04 ± 0.002
0	2	0	0.2	4.56 ± 0.128	3.43 ± 0.005	0.05 ± 0.002
0	2	0.1	0	4.38 ± 0.155	3.37 ± 0.004	0.06 ± 0.002
0	2	0.1	0.2	4.69 ± 0.176	3.50 ± 0.005	0.09 ± 0.002
0.5	0	0	0	1.31 ± 0.120	1.83 ± 0.004	0.00 ± 0. 000
0.5	0	0	0.2	2.63 ± 0.125	1.97 ± 0.007	0.00 ± 0. 000
0.5	0	0.1	0	2.38 ± 0.125	1.90 ± 0.008	0.00 ± 0. 000
0.5	0	0.1	0.2	2.69 ± 0.176	2.32 ± 0.009	0.00 ± 0. 000
0.5	0.5	0	0	2.81 ± 0.209	2.60 ± 0.007	0.00 ± 0. 000
0.5	0.5	0	0.2	3.25 ± 0.296	2.74 ± 0.005	0.00 ± 0. 000
0.5	0.5	0.1	0	3.19 ± 0.164	2.70 ± 0.006	0.00 ± 0. 000
0.5	0.5	0.1	0.2	3.50 ± 0.183	2.90 ± 0.008	0.00 ± 0. 000
0.5	1	0	0	3.81 ± 0.164	3.19 ± 0.006	0.05 ± 0.002
0.5	1	0	0.2	4.00 ± 0.204	3.38 ± 0.007	0.07 ± 0.003
0.5	1	0.1	0	4.13 ± 0.125	3.27 ± 0.005	0.10 ± 0.003
0.5	1	0.1	0.2	4.44 ± 0.157	3.39 ± 0.006	0.12 ± 0.002
0.5	2	0	0	4.56 ± 0.223	3.64 ± 0.005	0.15 ± 0.002
0.5	2	0	0.2	5.13 ± 0.155	3.72 ± 0.006	0.16 ± 0.002
0.5	2	0.1	0	5.31 ± 0.198	3.66 ± 0.006	0.18 ± 0.002
0.5	2	0.1	0.2	5.50 ± 0.129	3.81 ± 0.007	0.20 ± 0.003
1	0	0	0	2.75 ± 0.144	2.61 ± 0.006	0.00 ± 0. 000
1	0	0	0.2	3.19 ± 0.164	2.81 ± 0.006	0.00 ± 0. 000
1	0	0.1	0	3.31 ± 0.151	2.76 ± 0.006	0.00 ± 0. 000
1	0	0.1	0.2	3.56 ± 0.223	3.03 ± 0.013	0.00 ± 0. 000
1	0.5	0	0	4.19 ± 0.188	3.23 ± 0.005	0.08 ± 0.003
1	0.5	0	0.2	4.38 ± 0.180	3.41 ± 0.006	0.08 ± 0.003
1	0.5	0.1	0	4.44 ± 0.157	3.44 ± 0.004	0.10 ± 0.003
1	0.5	0.1	0.2	4.56 ± 0.182	3.53 ± 0.005	0.13 ± 0.003
1	1	0	0	4.50 ± 0.224	3.55 ± 0.005	0.11 ± 0.003
1	1	0	0.2	4.75 ± 0.112	3.65 ± 0.004	0.13 ± 0.002
1	1	0.1	0	4.81 ± 0.136	3.61 ± 0.007	0.14 ± 0.003
1	1	0.1	0.2	5.19 ± 0.136	3.68 ± 0.006	0.15 ± 0.003
1	2	0	0	5.81 ± 0.136	3.76 ± 0.005	0.21 ± 0.002
1	2	0	0.2	6.00 ± 0.158	3.90 ± 0.006	0.22 ± 0.003
1	2	0.1	0	6.19 ± 0.164	3.80 ± 0.006	0.24 ± 0.003
1	2	0.1	0.2	6.50 ± 0.129	3.99 ± 0.006	0.25 ± 0.003
2	0	0	0	4.44 ± 0.157	3.36 ± 0.004	0.06 ± 0.004
2	0	0	0.2	4.63 ± 0.125	3.47 ± 0.004	0.07 ± 0.003
2	0	0.1	0	4.75 ± 0.112	3.39 ± 0.006	0.08 ± 0.004
2	0	0.1	0.2	4.94 ± 0.143	3.58 ± 0.006	0.10 ± 0.003
2	0.5	0	0	4.69 ± 0.120	3.66 ± 0.005	0.18 ± 0.003
2	0.5	0	0.2	4.88 ± 0.180	3.80 ± 0.007	0.19 ± 0.003
2	0.5	0.1	0	5.25 ± 0.144	3.72 ± 0.004	0.21 ± 0.003
2	0.5	0.1	0.2	5.50 ± 0.129	3.90 ± 0.007	0.24 ± 0.004
2	1	0	0	6.06 ± 0.170	3.86 ± 0.004	0.22 ± 0.003
2	1	0	0.2	5.88 ± 0.180	3.89 ± 0.008	0.23 ± 0.003
2	1	0.1	0	5.75 ± 0.194	3.78 ± 0.007	0.25 ± 0.003
2	1	0.1	0.2	5.00 ± 0.183	3.91 ± 0.006	0.26 ± 0.003
2	2	0	0	4.00 ± 0.183	3.90 ± 0.005	0.25 ± 0.002
2	2	0	0.2	4.31 ± 0.120	3.89 ± 0.005	0.25 ± 0.003
2	2	0.1	0	4.56 ± 0.157	3.82 ± 0.006	0.27 ± 0.002
2	2	0.1	0.2	3.00 ± 0.183	3.71 ± 0.006	0.30 ± 0.004

### ANN modeling and evaluation

**Fig 3:**
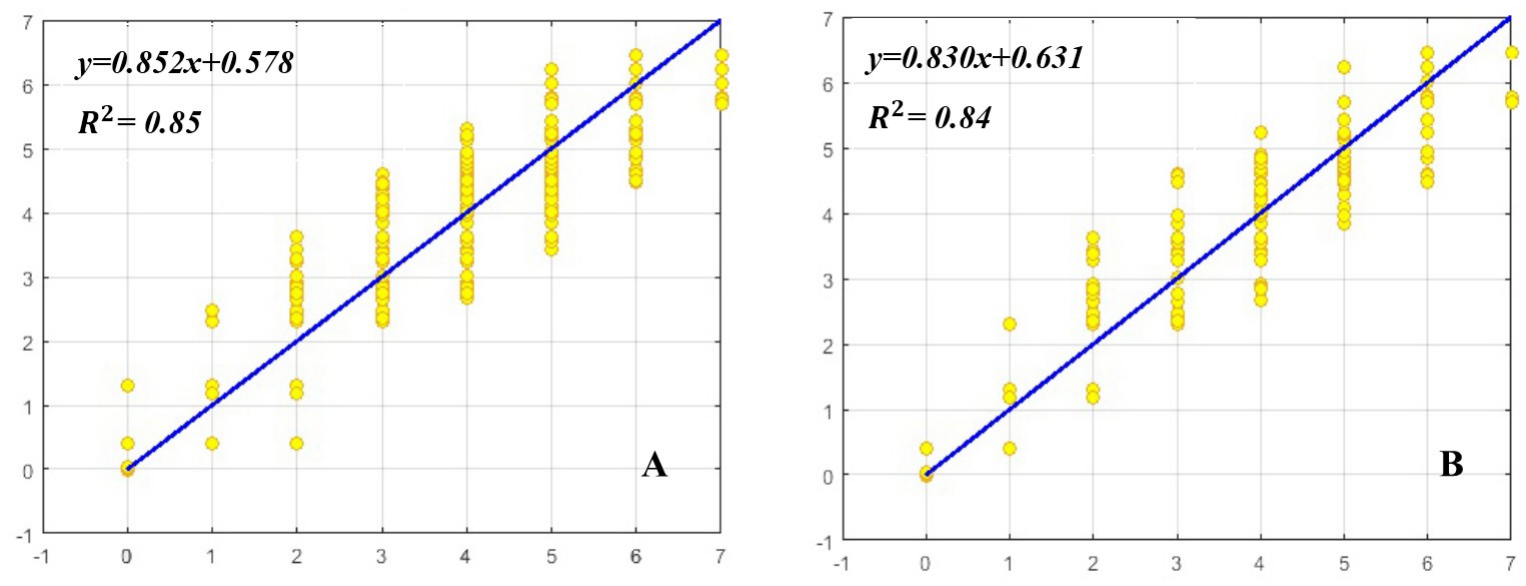
Scatter plot of predicted vs. observed values of shoot number (SN) obtained by MLP model. (A) Training set and (B) testing set.

**Fig 4:**
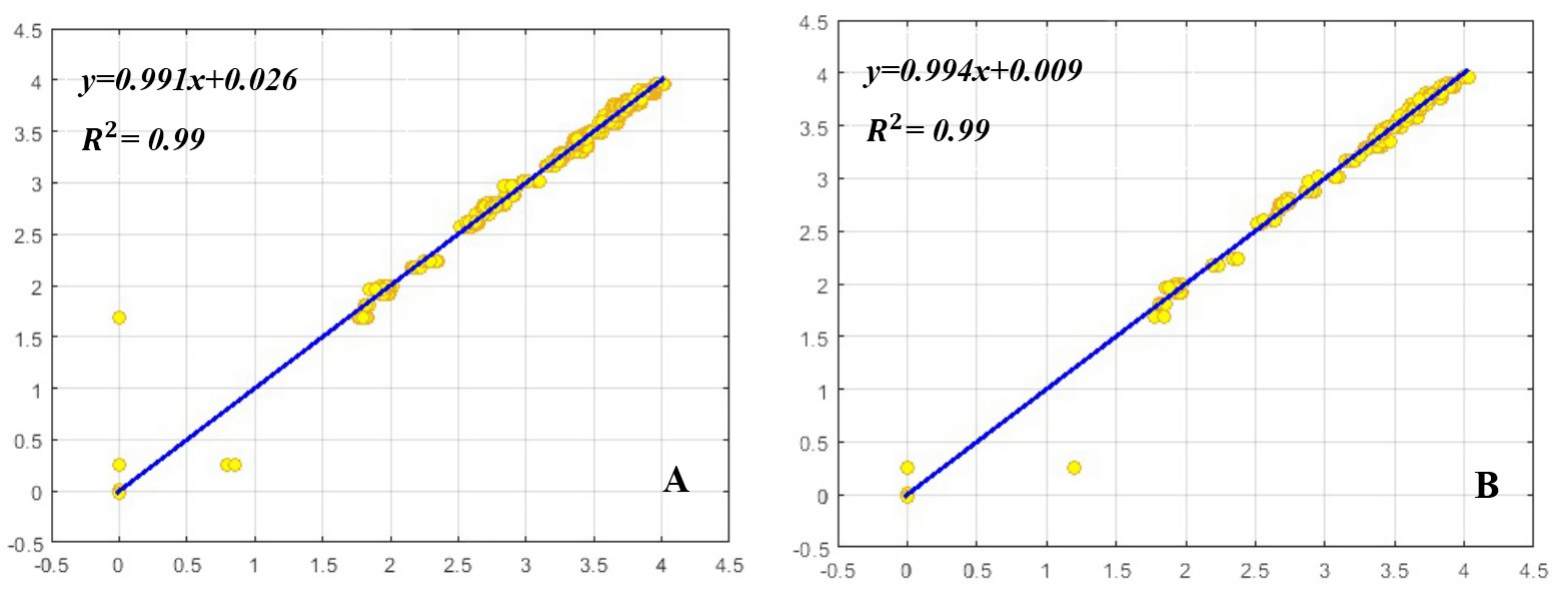
Scatter plot of model predicted vs. observed values of shoot length (SL) obtained by MLP model. (A) Training set and (B) testing set.

**Fig 5:**
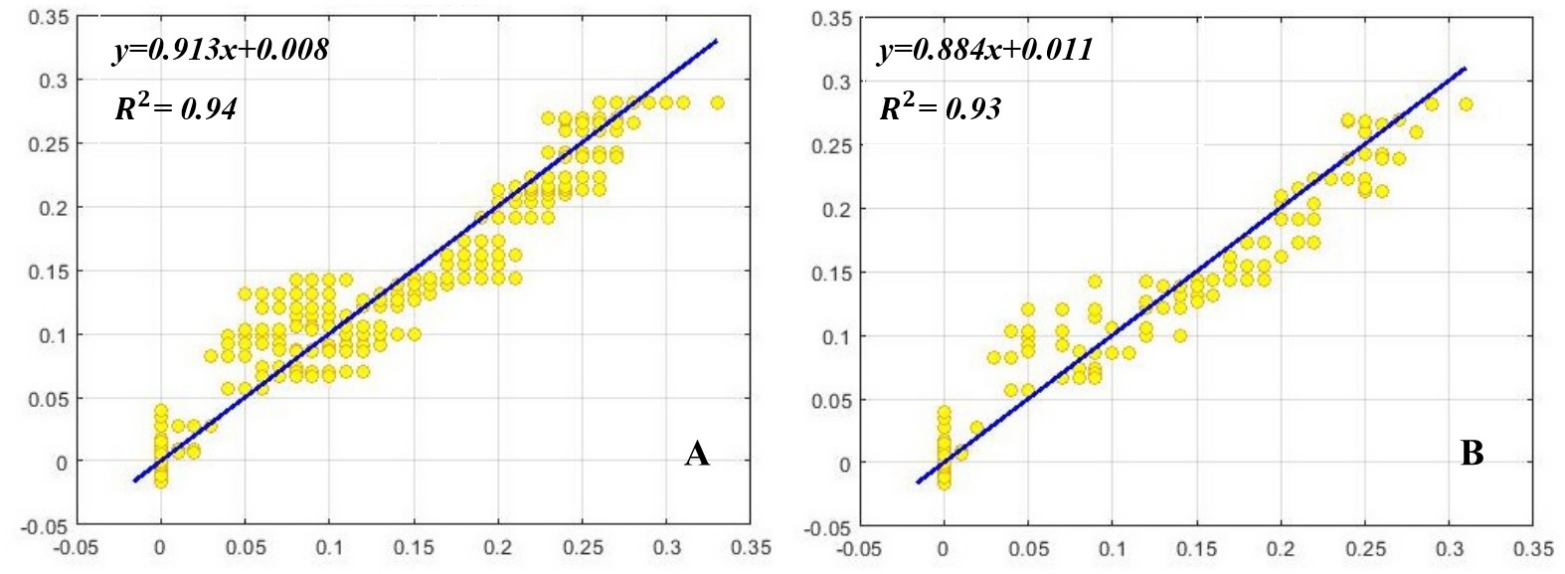
Scatter plot of model predicted vs. observed values of callus weight (CW) obtained by MLP model. (A) Training set and (B) testing set.

MLP was used to model and predict the effect of BA, Kin, NAA, and GA_3_ on shoot proliferation parameters including SN, SL, and CW of *E*. *cheiri*. R^2^, RMSE, and MBE of this model were presented in Table 2. Higher significant R^2^ value and lower RMSE and MBE values proved the model’s capability. The regression graphs (Figs 3–5) that presented this correlation, was efficient in predicting the outputs, and the values estimated by MLP were similar to the results of the experimental data (Table 2).

**Table 2:** Statistical information of MLP models for shoot number (SN), shoot length (SL), and callus weight (CW) of *E*. *cheiri* (training vs. testing values).

Item	Shoot number		Shoot length		Callus weight	
	Training	Testing	Training	Testing	Training	Testing
***R* Square**	0.85	0.84	0.99	0.99	0.94	0.93
**RMSE**	0.62	0.68	0.08	0.08	0.02	0.02
**MBE**	0.0002	-0.005	0.00009	-0.007	0.0003	0.001

### Model optimization

The final aim of this study was to optimize the MLP model by NSGA-II for providing accurate concentrations of PGRs and also obtain maximum SN, and SL and minimum CW. The optimal SN (7.12), SL (3.99 cm), and CW (0.21 g) can be obtained from a medium containing 1.41 mg.l^-1^ BA, 1.17 mg.l^-1^ KIN, 0.04 mg.l^-1^ NAA and 0.14 mg.l^-1^ GA_3_ (Table 3).

**Table 3:** The optimal values of hormonal combination as input items and predicted values of shoot number (SN), shoot length (SL), and callus weight (CW) by MLP-NSGA-II.

Input items				Output items		
BA (mg.l^-1^)	Kin (mg.l^-1^)	NAA (mg.l^-1^)	GA_3_ (mg.l^-1^)	Predicted SN	Predicted SL (cm)	Predicted CW (g)
1.41	1.17	0.04	0.14	7.12	3.99	0.21

### Sensitivity analyses

The importance of each input was evaluated through the VSR achieved for every output (SN, SL, and CW) (Table 4). The results of sensitivity analysis were indicated in (Table 4). Based on the sensitivity analysis, shoot number and callus weight were more sensitive to BA, followed by Kin, NAA and GA_3_ (Table 4). The most important factors which affected shoot length (SL), were BA followed by Kin, GA_3_ and NAA (Table 4). In contrary with SN and CW, SL was more sensitive to GA_3_ than NAA (Table 4).

**Table 4:** Ranking the importance of hormonal compositions on shooting proliferation parameters according to sensitivity analysis on the developed MLP model.

Output	Item	BA	Kin	NAA	GA_3_
**SN**	**VSR**	2.162	2.067	1.074	1.071
	**Rank**	1	2	3	4
**SL**	**VSR**	9.189	8.844	1.263	1.425
	**Rank**	1	2	4	3
**CW**	**VSR**	3.573	3.114	1.084	1.010
	**Rank**	1	2	3	4

### Validation experiment

**Fig 6:**
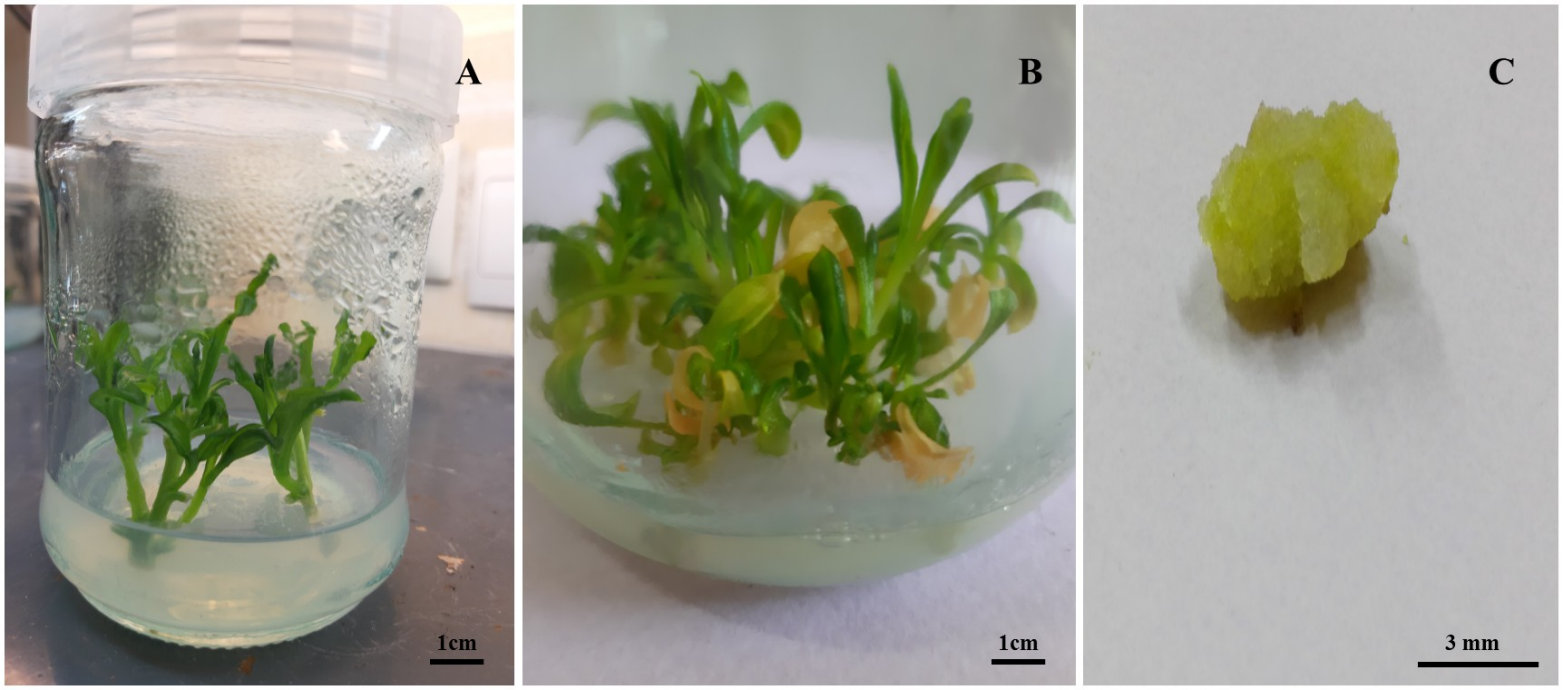
Validation experiment: (A) In vitro shoot regeneration from nodal segment explants of *E*.*cheiri* on MS medium with optimal values of hormonal combination containing 1.41 mg.l^-1^ BA, 1.17 mg.l^-1^ KIN, 0.04 mg.l^-1^ NAA and 0.14 mg.l^-1^ GA_3_, 30 days after culture. (B) In vitro development of multiple shoots after two months. (C) Callus induction in the predicted hormonal medium.

According to the validation experiment (Table 5), there was negligible difference between experimental validation data and predicted data via MLP-NSGAII. The predicted hormonal compositions via MLP-NSGAII resulted in 7.1 SN, 3.67 cm SL, and 0.19 g CW (Table 5) (Fig 6A–6C).

**Table 5:** Validation of the predicted data for shoot number (SN), shoot length (SL), and callus weight (CW). Values in each column represent means ± SE.

Treatment	SN	SL (cm)	CW (g)
Ideal point in NSGAII process	7.1 ± 0.31	3.67 ± 0.23	0.19 ± 0.01

## Discussion

The reliability and applicability of machine learning as one of the powerful computational approaches have been recently reviewed in different areas of plant science such as in vitro culture [4], plant breeding [27], stress phenotyping [28], and system biology [29]. Moreover, the accuracy of ANNs has been recently approved for modeling, prediction, and optimization of different in vitro culture systems such as sterilization [20, 30], seed germination [5, 31], callogenesis [32, 33], shoot proliferation [19, 34–36], somatic embryogenesis [37, 38], androgenesis [39], gene transformation [40, 41], and secondary metabolite production [42, 43].

There are many approaches for optimizing the culture medium for plant tissue culture, but there is not a universal protocol that can be used to modify a micropropagation medium for a large number of plants. Optimizing in vitro micropropagation as a multivariable and complex system is a highly tedious, expensive, and time-consuming process and traditional statistical methodology such as regression models alone are not reliable for approximation of these non-linear variables [4]. Therefore, there is a serious need for the application of new computational approaches such as ANNs to analyze and optimize this type of system more efficiently using fewer treatments [14, 20].

MLP model as one of the most popular types of ANNs, is employed in many micropropagation studies and contains three main parts: one input layer, one or more hidden layers and one output layer, which can be successfully employed for prediction, classification, signal processing and error filtering [14, 18]. Training and designing of ANN encounter to several problems. One of the most important problems is assigning the weights in ANN structure which demonstrates the direct effect on model performance [14]. The genetic algorithm is applied to find the optimal point of complex nonlinear functions in integrating with the artificial neural network that has a lot of advantages such as increasing the accuracy of ANN by updating the weights and bias values [14, 18]. Therefore, the hybridization of ANNs and multi-objective optimization algorithms can be considered as an accurate and reliable methodology for predicting and optimizing in vitro culture [5]. High coefficient of determination between observed and predicted values for both training and testing process indicated good performance of the models for the studied parameters [19]. The high efficiency of MLP in plant tissue culture has been shown by several studies. For instance, Arab et al. [22] employed MLP- GA for modeling and anticipating the optimal hormonal combinations in G × N15 vegetative rootstock proliferation. They reported the high accuracy of MLP-GA models (R^2^ > 0.81). Zhang et al. [44] used MLP for modeling and predicting organogenic callus production of melon and reported that MLP was able to accurately model and predict the system (R^2^ > 0.96). In another study, MLP was employed to model and predict in vitro root formation in grapevine [45]. They reported the high accuracy of MLP-GA model (R^2^> 0.78). However, GA as a single-objective algorithm cannot optimize multi-objective functions related to tissue culture problems simultaneously [19]. Therefore, a multi-objective evolutionary algorithms (MOEAs) has been required to optimize the outputs [20]. The main benefit of MOEAs is that they generate reasonably good approximations of the non-dominated frontier during a single run and in limited computational time [46, 47]. NSGAII as a multi-objective evolutionary algorithms generates a group of non-dominated solutions (identified as Pareto-optimal solutions) to find an equivalent solution between different objective functions and improves each of the objective functions without worsening other function values to guides the population towards the Pareto front [4]. In this study, CW had a negative effect on micropropagation due to the somaclonal variation and limitations of vascular system. Therefore, NSGAII algorithm was hybridized with MLP model to find the accurate concentration of hormonal compositions applied to obtain the maximum SN, SL as well as the minimum CW. Hesami et al. [20] applied MLP-NSGAII to optimize different types and concentrations of disinfectants and immersion time to minimize in vitro contamination and maximize the viability of Chrysanthemum explants. They reported the high accuracy of MLP- NSGAII model (R^2^> 0.94). In this work, we used MLP-NSGAII model to predict and optimize the hormonal combinations on shoot proliferation of *E*. *cheiri* and to achieve a new insights into improving in vitro culture. High coefficient of determination (0.84, 0.99 and 0.93 for SN, SL, and CW, respectively) between observed and predicted values for both training and testing processes showed that this method can be considered as an efficient method for analyzing and predicting *in vitro* growth condition for *E*. *cheiri* (Table 2). Therefore, validation experiments confirmed the results predicted by this method (Table 5).

Both the type and concentration of hormonal combinations play a critical role in plants’ proliferation, therefore each plant species needs a special concentration of hormones according to its internal hormones content [22]. Auxins and cytokinins, as the major plant hormones affect the cell division and multiplication of plant tissues [32]. Among them, cytokinins are believed to be the most important plant hormone responsible for stimulation of cell divisions and shoot proliferation [48]. In some cases, combination of cytokinins have proved more effective leading to increased proliferation rate [48]. This is in the line with our results which showed that increasing the concentration of two cytokinins BA and KIN up to 2 mg.l^-1^ and 1 mg.l^-1^ respectively, led to enhanced shooting parameters (Table 1). Further concentrations of cytokinins’ combination reduced the shoot production and increased callus formation. Nowakowska et al. [48] in the same way reported that too high cytokinin concentrations in the medium inhibited shoot multiplication and negatively affected shoot length. Many other researchers have also reported similar results [49, 50]. Auxins are the other vital plant hormone that triggers many plant activities, such as root and shoot production, stimulation of callus cell divisions, and inducing apical domination [51]. Low contents of auxins along with high concentrations of cytokinins affect cell divisions and are responsible for the *in vitro* regeneration and shoot proliferation [52, 53]. Auxins are considered to exhibit synergistic, antagonistic and additive interactions with cytokinins depending on the plant species and tissue type in the regulation of physiological responses [54]. In fact cytokinin acts as a positive regulator of auxin biosynthesis and auxin as a negative regulator of cytokinin biosynthesis and both are controlled by a homeostatic regulatory mechanism [55]. NAA is the only auxin that does not require active uptake to easily pass through the plasma membrane into cells and has a synergistic/additive effect on shoot proliferation [56]. In our experiment the combined use of BA and NAA caused higher plant shooting than when using them alone (Table 1). These results are in line with the findings of other species such as G × N15 rootstock [22], *Daphne mezereum* ‘Alba’ [48], Chinese ginger (*Boesenbergia rotunda*) [57], *Cassia angustifolia* [58], *Magnolia sirindhorniae* [59] and *Santolina canescens* [60]. However, high concentrations of hormonal combinations decrease shoot length and shooting rate and conversely increase callus development [22, 61] which confirms our findings in *E*. *cheiri* (Table 1).

Gibberellins are other important plant growth regulators which its most active and popular member, gibberellic acid (GA_3_) plays important roles in plant development, seed germination, shoot elongation and flower induction [48, 62, 63]. Combinations of cytokinin and GA_3_ were found to be the best for both shoot multiplication and shoot elongation [59, 64], which our results are in accordance with these findings (Table 1). Contrary reports have shown less shoots when BA was combined with GA_3_ compared to with BA treatment alone [48, 59, 65, 66]. The media not supplemented with cytokinins had a high GA_3_ absorption, suggesting that the presence of cytokinins could negatively affect explant GA_3_ uptake [67]. High GA_3_ concentration in combination with low BAP concentration was necessary for high shoot elongation in kiwifruit (*Actinidia arguta*) [63]. Moreover, a number of researches have shown that combination of GA_3_ and NAA indicate a positive effect on *in vitro* shoot multiplication of plants [6, 56, 68]. Optimization of auxin concentration is regarded as a key factor in controlling plantlet height [45]. Zhang et al. [69] suggest that the shoot length of potato explants was increased when higher concentrations of IAA were used. However, the effect of IAA is improved by the addition of GA_3_ [69]. Furthermore, combination of GA_3_ and NAA concentrations in a number of plants demonstrated increased shoot length [70, 71]. Several studies have shown that high callus production also occurs due to increased concentrations of auxins in plants [72, 73]. *In vitro* plant regeneration is mainly dependent on exogenous and endogenous phytohormones [4, 74]. Due to the high callus production rate of *E*. *cheiri*, it is considered that in addition to external growth regulators applied, the amount of internal auxin in this plant is also possibly high. Based on the results of sensitivity analysis (Table 4), BA was found to be superior than Kin, in terms of the overall number and length of shoots produced per explant, which is in agreement with findings of Akbas et al. [75]. Finally, according to the validation experiment, MLP-NSGAII as a new computational algorithm in analyzing data derived from *in vitro* culture, could be able to propose the optimal level of hormonal combinations to achieve the most appropriate results of the investigated parameters.

## Conclusion

Plant tissue culture is a complex process in which many diverse factors are involved. In order to achieve an optimum protocol, several treatments with multiple replications and numerous trials and errors are designed, which have proved to be very costly and time consuming. Recently some computational techniques such as ANN models have been suggested to analyze and optimize multi objective processes. In this study for the first time, MLP-NSGAII was implemented for *E*. *cheiri* as a new computational tool for optimizing and predicting *in vitro* shoot proliferation. Based on the results, MLP-NSGAII is an efficient method for modeling and optimizing the hormonal combination for plant *in vitro* culture. We anticipate that it may be applicable for other plant species and other features such as mineral compounds, light and temperature as well. In future studies, MLP should be compared with other machine learning algorithms.

## Supporting information

S1 Data (XLS)

## References

[1] Polatschek A. Revision der gattung *Erysimum* (Cruciferae), Teil 2: georgien, armenien, azerbaidzan, türkei, syrien, libanon, Israel, Jordanien, irak, Iran, Afghanistan. Annalen des Naturhistorischen Museums in Wien Serie B für Botanik und Zoologie. 2010:369–497.

[2] Moazzeni H, Zarre S, Pfeil BE, Bertrand YJ, German DA, Al-Shehbaz IA, et al. Phylogenetic perspectives on diversification and character evolution in the species-rich genus *Erysimum* (Erysimeae; Brassicaceae) based on a densely sampled ITS approach. Bot J Linn. Soc. 2014;175(4):497–522.

[3] Mosleh G, Badr P, Azadi A, Abolhassanzadeh Z, Hosseini SV. Wallflower (*Erysimum cheiri* (L.) Crantz) from past to future. Res J Pharm. 2019;6(2):85–95.

[4] Hesami M, Jones AMP. Application of artificial intelligence models and optimization algorithms in plant cell and tissue culture. Appl Microbiol Biotechnol. 2020:1–37. doi: 10.1007/s00253-020-10888-2 32984921

[5] Hesami M, Pepe M, Monthony AS, Baiton A, Jones AMP. Modeling and optimizing in vitro seed germination of industrial hemp (*Cannabis sativa* L.). Ind Crops Prod. 2021;170:113753.

[6] Pati PK, Rath SP, Sharma M, Sood A, Ahuja PS. In vitro propagation of rose—a review. Biotechnol Adv. 2006;24(1):94–114. doi: 10.1016/j.biotechadv.2005.07.001 16169183

[7] Arab MM, Yadollahi A, Shojaeiyan A, Shokri S, Ghojah SM. Effects of nutrient media, different cytokinin types and their concentrations on in vitro multiplication of G× N15 (hybrid of almond× peach) vegetative rootstock. J Genet Eng Biotechnol. 2014;12(2):81–7.

[8] Hesami M, Daneshvar MH, Yoosefzadeh-Najafabadi M, Alizadeh M. Effect of plant growth regulators on indirect shoot organogenesis of *Ficus religiosa* through seedling derived petiole segments. J Genet Eng Biotechnol. 2018;16(1):175–80. 30647720 10.1016/j.jgeb.2017.11.001 PMC6296569

[9] Ghosh A, Igamberdiev AU, Debnath SC. Thidiazuron-induced somatic embryogenesis and changes of antioxidant properties in tissue cultures of half-high blueberry plants. Sci Rep. 2018;8(1):1–11. 30451961 10.1038/s41598-018-35233-6 PMC6242952

[10] Ikeuchi M, Sugimoto K, Iwase A. Plant callus: mechanisms of induction and repression. Plant Cell. 2013;25(9):3159–73. doi: 10.1105/tpc.113.116053 24076977 PMC3809525

[11] Amoo SO, Van Staden J. Influence of plant growth regulators on shoot proliferation and secondary metabolite production in micropropagated *Huernia hystrix*. Plant Cell Tissue Organ Cult. 2013;112(2):249–56.

[12] Gago J, Martínez-Núñez L, Landín M, Gallego P. Artificial neural networks as an alternative to the traditional statistical methodology in plant research. J Plant Physiol. 2010b;167(1):23–7. 19716625 10.1016/j.jplph.2009.07.007

[13] Niazian M, Sadat-Noori SA, Abdipour M. Modeling the seed yield of Ajowan (*Trachyspermum ammi* L.) using artificial neural network and multiple linear regression models. Ind Crops Prod. 2018;117:224–34.

[14] Silva JCF, Teixeira RM, Silva FF, Brommonschenkel SH, Fontes EP. Machine learning approaches and their current application in plant molecular biology: A systematic review. Plant Sci. 2019;284:37–47. doi: 10.1016/j.plantsci.2019.03.020 31084877

[15] Jafari M, Shahsavar A. The application of artificial neural networks in modeling and predicting the effects of melatonin on morphological responses of citrus to drought stress. PloS One. 2020;15(10):e0240427. doi: 10.1371/journal.pone.0240427 33052940 PMC7556499

[16] Jamshidi S, Yadollahi A, Ahmadi H, Arab M, Eftekhari M. Predicting in vitro culture medium macro-nutrients composition for pear rootstocks using regression analysis and neural network models. Front Plant Sci. 2016;7:274. doi: 10.3389/fpls.2016.00274 27066013 PMC4809900

[17] Arab MM, Yadollahi A, Shojaeiyan A, Ahmadi H. Artificial neural network genetic algorithm as powerful tool to predict and optimize in vitro proliferation mineral medium for G× N15 rootstock. Front Plant Sci. 2016;7:1526. doi: 10.3389/fpls.2016.01526 27807436 PMC5069296

[18] Salehi M, Farhadi S, Moieni A, Safaie N, Ahmadi H. Mathematical modeling of growth and paclitaxel biosynthesis in *Corylus avellana* cell culture responding to fungal elicitors using multilayer perceptron-genetic algorithm. Front Plant Sci. 2020;11. 10.3389/fpls.2020.01148 PMC7432144 32849706

[19] Hesami M, Naderi R, Tohidfar M. Modeling and optimizing medium composition for shoot regeneration of Chrysanthemum via radial basis function-non-dominated sorting genetic algorithm-II (RBF-NSGAII). Sci Rep. 2019a;9(1):1–11. 31796784 10.1038/s41598-019-54257-0 PMC6890634

[20] Hesami M, Naderi R, Tohidfar M. Modeling and optimizing in vitro sterilization of chrysanthemum via multilayer perceptron-non-dominated sorting genetic algorithm-II (MLP-NSGAII). Front Plant Sci. 2019c;10:282. 30923529 10.3389/fpls.2019.00282 PMC6426794

[21] Hesami M, Naderi R, Tohidfar M, Yoosefzadeh-Najafabadi M. Application of adaptive neuro-fuzzy inference system-non-dominated sorting genetic Algorithm-II (ANFIS-NSGAII) for modeling and optimizing somatic embryogenesis of Chrysanthemum. Front Plant Sci. 2019b;10:869. 31333705 10.3389/fpls.2019.00869 PMC6624437

[22] Arab MM, Yadollahi A, Ahmadi H, Eftekhari M, Maleki M. Mathematical modeling and optimizing of in vitro hormonal combination for G× N15 vegetative rootstock proliferation using Artificial Neural Network-Genetic Algorithm (ANN-GA). Front Plant Sci. 2017;8:1853. doi: 10.3389/fpls.2017.01853 29163583 PMC5672016

[23] Eftekhari M, Yadollahi A, Ahmadi H, Shojaeiyan A, Ayyari M. Development of an artificial neural network as a tool for predicting the targeted phenolic profile of grapevine (*Vitis vinifera*) foliar wastes. Front Plant Sci. 2018;9:837. 29971086 10.3389/fpls.2018.00837 PMC6018394

[24] Murashige T, Skoog F. A revised medium for rapid growth and bio assays with tobacco tissue cultures. Physiol Plant. 1962;15(3):473–97.

[25] Wanas N, Auda G, Kamel MS, Karray F, editors. On the optimal number of hidden nodes in a neural network. Conference Proceedings IEEE Canadian Conference on Electrical and Computer Engineering (Cat No 98TH8341); 1998: IEEE.

[26] Ahmadi H, Rodehutscord M. Application of artificial neural network and support vector machines in predicting metabolizable energy in compound feeds for pigs. Front Nutr. 2017;4:27. doi: 10.3389/fnut.2017.00027 28713814 PMC5491901

[27] van Dijk ADJ, Kootstra G, Kruijer W, de Ridder D. Machine learning in plant science and plant breeding. Iscience. 2021;24(1):101890. doi: 10.1016/j.isci.2020.101890 33364579 PMC7750553

[28] Singh A, Ganapathysubramanian B, Singh AK, Sarkar S. Machine learning for high-throughput stress phenotyping in plants. Trends Plant Sci. 2016;21(2):110–24. doi: 10.1016/j.tplants.2015.10.015 26651918

[29] Hesami M, Alizadeh M, Jones AMP, Torkamaneh D. Machine learning: its challenges and opportunities in plant system biology. Appl Microbiol Biotechnol. 2022:1–24. 35575915 10.1007/s00253-022-11963-6

[30] Pepe M, Hesami M, Jones AMP. Machine Learning-Mediated Development and Optimization of Disinfection Protocol and Scarification Method for Improved In Vitro Germination of Cannabis Seeds. Plants. 2021;10(11):2397. doi: 10.3390/plants10112397 34834760 PMC8619272

[31] Aasim M, Katırcı R, Akgur O, Yildirim B, Mustafa Z, Nadeem MA, et al. Machine learning (ML) algorithms and artificial neural network for optimizing in vitro germination and growth indices of industrial hemp (*Cannabis sativa* L.). Ind Crops Prod. 2022;181:114801.

[32] Munasinghe SP, Somaratne S, Weerakoon SR, Ranasinghe C. Prediction of chemical composition for callus production in *Gyrinops walla* Gaetner through machine learning. Inf Proc Agr. 2020;7(4):511–22.

[33] Hesami M, Jones AMP. Modeling and optimizing callus growth and development in *Cannabis sativa* using random forest and support vector machine in combination with a genetic algorithm. Appl Microbiol Biotechnol. 2021;105(12):5201–12. 34086118 10.1007/s00253-021-11375-y

[34] Kirtis A, Aasim M, Katırcı R. Application of artificial neural network and machine learning algorithms for modeling the in vitro regeneration of chickpea (*Cicer arietinum* L.). Plant Cell Tissue Organ Cult. 2022:1–12.

[35] Hesami M, Condori-Apfata JA, Valderrama Valencia M, Mohammadi M. Application of artificial neural network for modeling and studying in vitro genotype-independent shoot regeneration in wheat. Appl Sci. 2020;10(15):5370.

[36] Pepe M, Hesami M, Small F, Jones AMP. Comparative analysis of machine learning and evolutionary optimization algorithms for precision micropropagation of *Cannabis sativa*: Prediction and validation of in vitro shoot growth and development based on the optimization of light and carbohydrate sources. Front Plant Sci. 2021;12. 10.3389/fpls.2021.757869 PMC8566924 34745189

[37] Hesami M, Naderi R, Tohidfar M. Introducing a hybrid artificial intelligence method for high-throughput modeling and optimizing plant tissue culture processes: the establishment of a new embryogenesis medium for chrysanthemum, as a case study. Appl Microbiol Biotechnol. 2020;104(23):10249–63. doi: 10.1007/s00253-020-10978-1 33119796

[38] Niazian M, Sadat-Noori SA, Abdipour M, Tohidfar M, Mortazavian SMM. Image processing and artificial neural network-based models to measure and predict physical properties of embryogenic callus and number of somatic embryos in ajowan (*Trachyspermum ammi* (L.) Sprague). In Vitro Cell Develop Biol Plant. 2018b;54(1):54–68.

[39] Niazian M, Shariatpanahi ME, Abdipour M, Oroojloo M. Modeling callus induction and regeneration in an anther culture of tomato (*Lycopersicon esculentum* L.) using image processing and artificial neural network method. Protoplasma. 2019;256(5):1317–32. 31055656 10.1007/s00709-019-01379-x

[40] Hesami M, Alizadeh M, Naderi R, Tohidfar M. Forecasting and optimizing Agrobacterium-mediated genetic transformation via ensemble model-fruit fly optimization algorithm: A data mining approach using chrysanthemum databases. PloS One. 2020;15(9):e0239901. doi: 10.1371/journal.pone.0239901 32997694 PMC7526930

[41] Niedbała G, Niazian M, Sabbatini P. Modeling agrobacterium-mediated gene transformation of tobacco (*Nicotiana tabacum*)—a model plant for gene transformation studies. Front Plant Sci. 2021;12. 10.3389/fpls.2021.695110 PMC8370025 34413865

[42] Salehi M, Farhadi S, Moieni A, Safaie N, Hesami M. A hybrid model based on general regression neural network and fruit fly optimization algorithm for forecasting and optimizing paclitaxel biosynthesis in *Corylus avellana* cell culture. Plant Methods. 2021;17(1):1–13. 33546685 10.1186/s13007-021-00714-9 PMC7866739

[43] Farhadi S, Salehi M, Moieni A, Safaie N, Sabet MS. Modeling of paclitaxel biosynthesis elicitation in *Corylus avellana* cell culture using adaptive neuro-fuzzy inference system-genetic algorithm (ANFIS-GA) and multiple regression methods. PloS One. 2020;15(8):e0237478. 32853208 10.1371/journal.pone.0237478 PMC7451515

[44] Zhang Q, Deng D, Dai W, Li J, Jin X. Optimization of culture conditions for differentiation of melon based on artificial neural network and genetic algorithm. Sci Rep. 2020;10(1):1–8. 32103071 10.1038/s41598-020-60278-x PMC7044330

[45] Gago J, Landín M, Gallego PP. A neurofuzzy logic approach for modeling plant processes: A practical case of in vitro direct rooting and acclimatization of *Vitis vinifera* L. Plant Sci. 2010a;179(3):241–9.

[46] Anagnostopoulos KP, Mamanis G. A portfolio optimization model with three objectives and discrete variables. Comput Oper Res. 2010;37(7):1285–97.

[47] Jamshidi S, Yadollahi A, Arab MM, Soltani M, Eftekhari M, Shiri J. High throughput mathematical modeling and multi-objective evolutionary algorithms for plant tissue culture media formulation: Case study of pear rootstocks. PloS One. 2020;15(12):e0243940. doi: 10.1371/journal.pone.0243940 33338074 PMC7748151

[48] Nowakowska K, Pacholczak A, Tepper W. The effect of selected growth regulators and culture media on regeneration of *Daphne mezereum* L.‘Alba’. Rend Fis Acc Lincei. 2019;30(1):197–205.

[49] Shekafandeh A. The effects of pH levels and plant growth regulators on in vitro regeneration of Almond (*Prunus dulcis* Mill.). World Appl Sci J. 2010;8(11):1322–6.

[50] Ahmad N, Faisal M, Anis M. Role of PGR on in vitro shoot propagation in *Cyamopsis tetragonoloba* L.(Taub.): a drought tolerant grain legume. Rend Fis Acc Lincei. 2013;24(1):7–12.

[51] Singh R, Kumar S, Kalia S, Sharma S, Kalia RK. Recent advances in understanding the role of growth regulators in plant growth and development in vitro—III. Inhibitors of growth regulators. Indian For. 2016;142(11):1065–72.

[52] Su Y-H, Liu Y-B, Zhang X-S. Auxin–cytokinin interaction regulates meristem development. Mol Plant. 2011;4(4):616–25. doi: 10.1093/mp/ssr007 21357646 PMC3146736

[53] Fatima N, Ahmad N, Anis M. Enhanced in vitro regeneration and change in photosynthetic pigments, biomass and proline content in *Withania somnifera* L.(Dunal) induced by copper and zinc ions. Plant Physiol Biochem. 2011;49(12):1465–71. 22078385 10.1016/j.plaphy.2011.08.011

[54] Coenen C, Lomax TL. Auxin—cytokinin interactions in higher plants: old problems and new tools. Trends Plant Sci. 1997;2(9):351–6. doi: 10.1016/S1360-1385(97)84623-7 11540614

[55] Jones B, Gunnerås SA, Petersson SV, Tarkowski P, Graham N, May S, et al. Cytokinin regulation of auxin synthesis in Arabidopsis involves a homeostatic feedback loop regulated via auxin and cytokinin signal transduction. Plant Cell. 2010;22(9):2956–69. doi: 10.1105/tpc.110.074856 20823193 PMC2965550

[56] Nordström A, Tarkowski P, Tarkowska D, Norbaek R, Åstot C, Dolezal K, et al. Auxin regulation of cytokinin biosynthesis in *Arabidopsis thaliana*: a factor of potential importance for auxin–cytokinin-regulated development. Proc Natl Acad Sci. 2004;101(21):8039–44. 15146070 10.1073/pnas.0402504101 PMC419553

[57] Yusuf NA, Annuar MS, Khalid N. Rapid micropropagation of *Boesenbergia rotunda* (L.) Mansf. Kulturpfl. (a valuable medicinal plant) from shoot bud explants. Afr J Biotechnol. 2011;10(7):1194–9.

[58] Siddique I, Bukhari NAW, Perveen K, Siddiqui I. Influence of plant growth regulators on in vitro shoot multiplication and plantlet formation in *Cassia angustifolia* Vahl. Braz Arch Biol Technol. 2015;58(5):686–91.

[59] Cui Y, Deng Y, Zheng K, Hu X, Zhu M, Deng X, et al. An efficient micropropagation protocol for an endangered ornamental tree species (*Magnolia sirindhorniae* Noot. & Chalermglin) and assessment of genetic uniformity through DNA markers. Sci Rep. 2019;9(1):1–10. 31270420 10.1038/s41598-019-46050-w PMC6610120

[60] Casado J, Navarro M, Utrilla M, Martinez A, Jimenez J. Micropropagation of *Santolina canescens* Lagasca and in vitro volatiles production by shoot explants. Plant Cell Tissue Organ Cult. 2002;69(2):147–53.

[61] Phulwaria M, Patel AK, Rathore JS, Ram K, Shekhawat N. An improved micropropagation and assessment of genetic stability of micropropagated *Salvadora oleoides* using RAPD and ISSR markers. Acta Physiolo Plant. 2014;36(5):1115–22.

[62] Zhang X, Wu Z, Huang C. Effects of gibberellin mutations on in vitro shoot bud regeneration of Arabidopsis. Afr J Biotechnol. 2008;7(22).

[63] Arteta T, Hameg R, Landin M, Gallego P, Barreal M. Neural networks models as decision-making tool for in vitro proliferation of hardy kiwi. Eur J Hortic Sci. 2018;83:259–65.

[64] Kavand S, Kermani MJ, Haghnazari A, Khosravi P, Azimi MR. Micropropagation and medium-term conservation of *Rosa pulverulenta*. Acta Sci Agron. 2011;33:297–301.

[65] Nagori R, Purohit S. In vitro plantlet regeneration in *Annona squamosa* through direct shoot bud differentiation on hypocotyl segments. Sci Hortic. 2004;99(1):89–98.

[66] Sabeti M, Zarghami R, Zadeh M. Effects of explants and growth regulators on callogenesis and somatic embryogenesis of Agria potato cultivar. Int J Agr. 2013;3(3):213–21.

[67] Moncaleán P, Cañal MJ, Fernández H, Fernández B, Rodríguez A. Nutritional and gibberellic acid requirements in kiwifruit vitroponic cultures. In Vitro Cell Dev Biol Plant. 2003;39(1):49–55.

[68] Roest S, Bokelmann G. Vegetative propagation of *Solanum tuberosum* L. in vitro. Potato Res. 1976;19(2):173–8.

[69] Zhang Z, Zhou W, Li H. The role of GA, IAA and BAP in the regulation of in vitro shoot growth and microtuberization in potato. Acta Physiol Plant. 2005;27(3):363–9.

[70] Badoni A, Chauhan J. Potato seed production of Cultivar Kufri Himalini, in vitro. Stem Cell. 2010;1(1):7–10.

[71] Webb KJ, Osifo EO, Henshaw GG. Shoot regeneration from leaflet discs of six cultivars of potato (*Solanum tuberosum* subsp. tuberosum). Plant Sci Lett. 1983;30(1):1–8.

[72] George EF, Hall MA, De Klerk G-J. Plant propagation by tissue culture: volume 1. the background: Springer Science & Business Media; 2007.

[73] Yaseen M, Ahmad T, Abbasi NA, Hafiz IA. Assessment of apple rootstocks M 9 and M 26 for in vitro rooting potential using different carbon sources. Pak J Bot. 2009;41(2):769–81.

[74] Kumari A, Baskaran P, Plačková L, Omámiková H, Nisler J, Doležal K, et al. Plant growth regulator interactions in physiological processes for controlling plant regeneration and in vitro development of *Tulbaghia simmleri*. J Plant Physiol. 2018;223:65–71. 29505949 10.1016/j.jplph.2018.01.005

[75] Akbas F, Isikalan Ç, Namli S, Ak BE. Effect of plant growth regulators on in vitro shoot multiplication of *Amygdalus communis* L. cv. Yaltsinki. Afr J Biotechnol. 2009;8(22).

